# Supplementary material for: Lethal Borna disease virus 1 infections of humans and animals – in-depth molecular epidemiology and phylogeography
Source: Nat Commun. 2024 Sep 10;15:7908. doi: 10.1038/s41467-024-52192-x (PMC11387626; doi:10.1038/s41467-024-52192-x)
Supplement: Supplementary file 1 — Supplementary Information [file 41467_2024_52192_MOESM1_ESM.pdf]

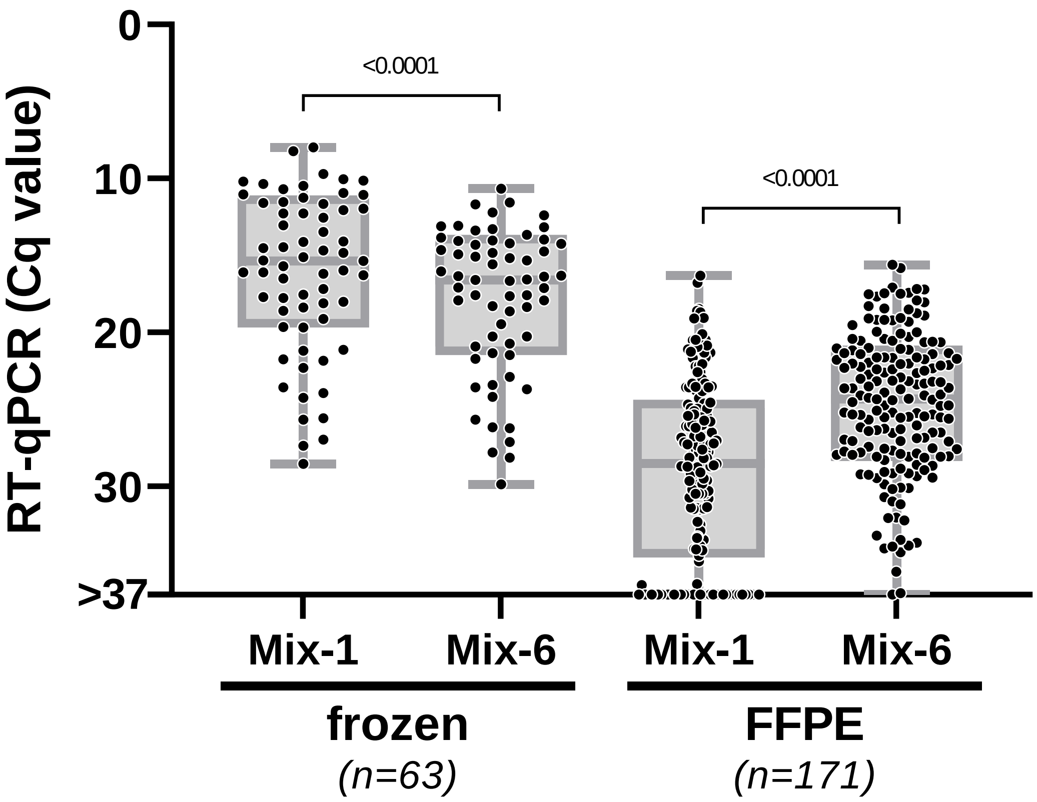

Supplementary Figure 1. Impact of sample quality on RT-qPCR sensitivity. Fresh-frozen or formalin-fixed paraffin-embedded (FFPE) brain samples from BoDV-1-infected domestic mammals, humans and shrews were analysed with BoDV-1-specific RT-qPCR assays Mix-1 and Mix-6. Mix-1 (amplicon size: 162 base pairs) targets the P gene. Mix-6 (75 base pairs) targets the M gene.

Statistical analysis was performed using paired Student´s t-test for comparison of Mix-1 and Mix-6 RT-qPCRs within one sample type. Numbers above horizontal lines represent *P* values. $P < 0.05$ is considered to indicate significant differences between the RT-qPCR assays. Box plot: centre line = median; box limits = upper and lower quartiles; whiskers = minimum and maximum.

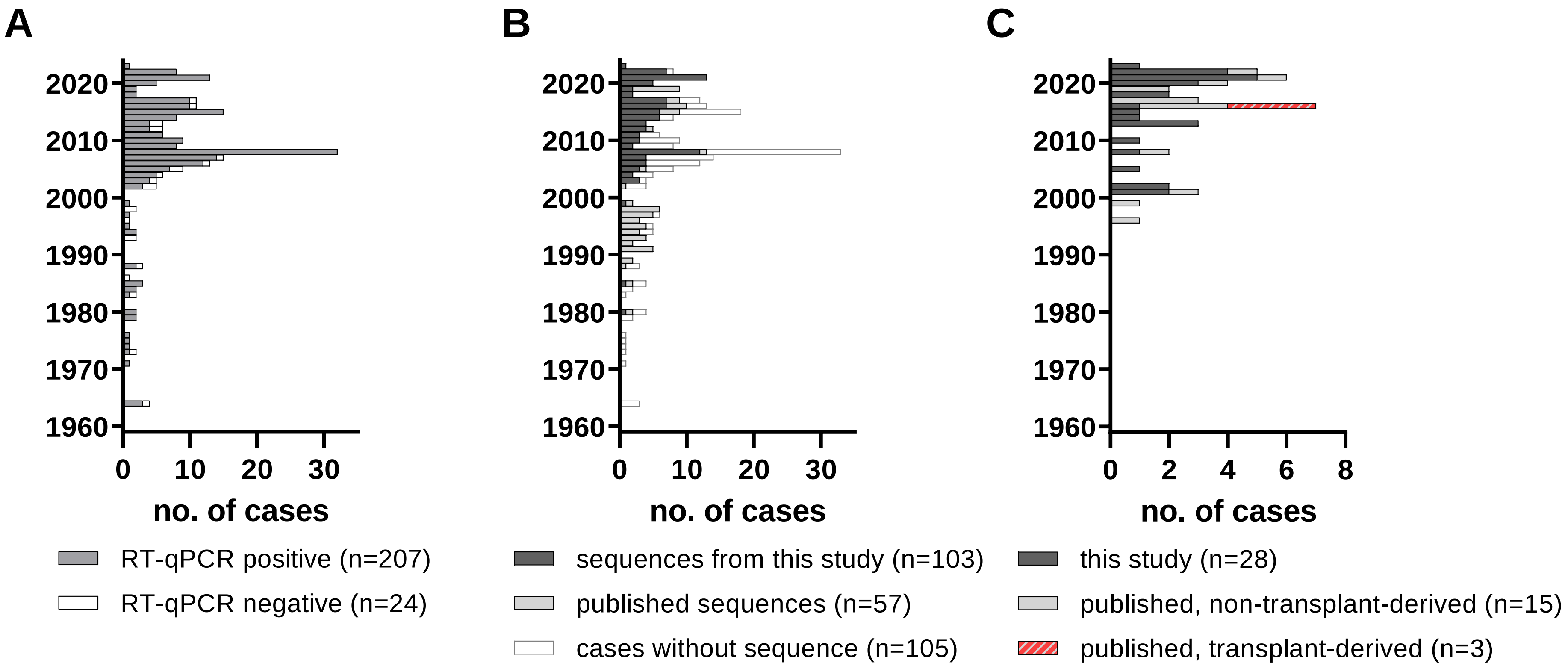

Supplementary Figure 2. Year of origin of analysed samples and confirmed BoDV-1 infections in this study. A) Confirmed or suspected BoDV-1 infections in domestic mammals submitted and analysed as part of this study (n=231). **B)** BoDV-1 infections in domestic mammals confirmed by RT-qPCR and/or sequencing during this and previous studies (n=265; including BoDV-1 laboratory strains originating from domestic mammals; see Table 1); **C)** Human BoDV-1 infections confirmed by RT-qPCR and/or sequencing during this and previous studies (n=46; see Table 1).

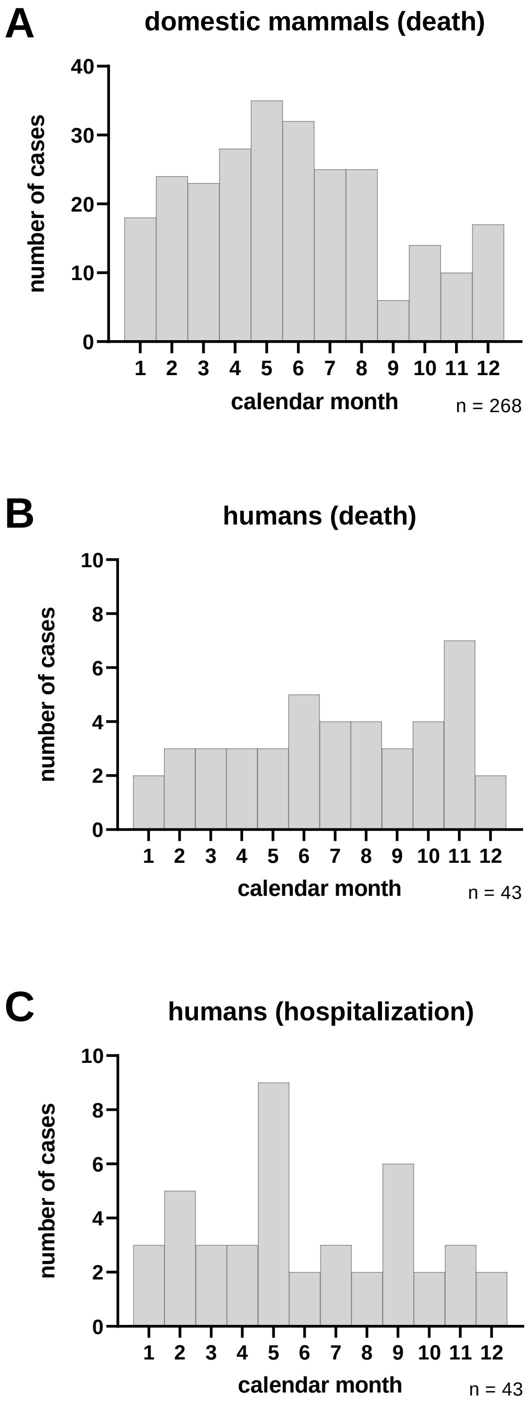

Supplementary Figure 3. Seasonal distribution of confirmed BoDV-1 infections in domestic mammals and humans. **A)** Time of death of domestic mammals. **B)** Time of death and **C)** time of hospitalization of BoDV-1-infected human patients. BoDV-1 infections confirmed in this and previously published studies are presented. Three human patients with solid organ transplant-derived infection [1] were excluded from this analysis, as they represent iatrogenic infections that are assumed to not follow the same epidemiologic pattern as natural infections.

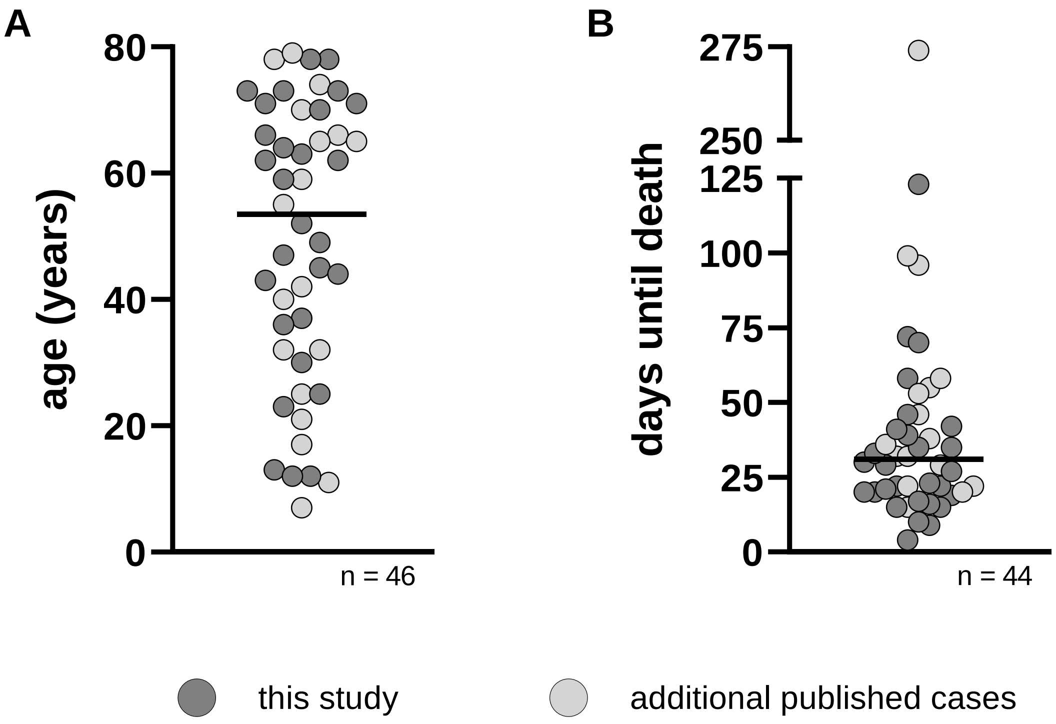

Supplementary Figure 4. Characteristics of patients with confirmed BoDV-1-infection (n=46) in this and previous studies. A) Age of patients at the time of death. **B)** Reported duration from initial hospitalization to death. Two patients of the previously published solid organ transplant cluster are not included in this panel. The liver recipient survived the acute disease whereas the donor died peracutely with his death not being unequivocally attributable to his BoDV-1 infection [1]. Horizontal lines represent the median of the respective data set.

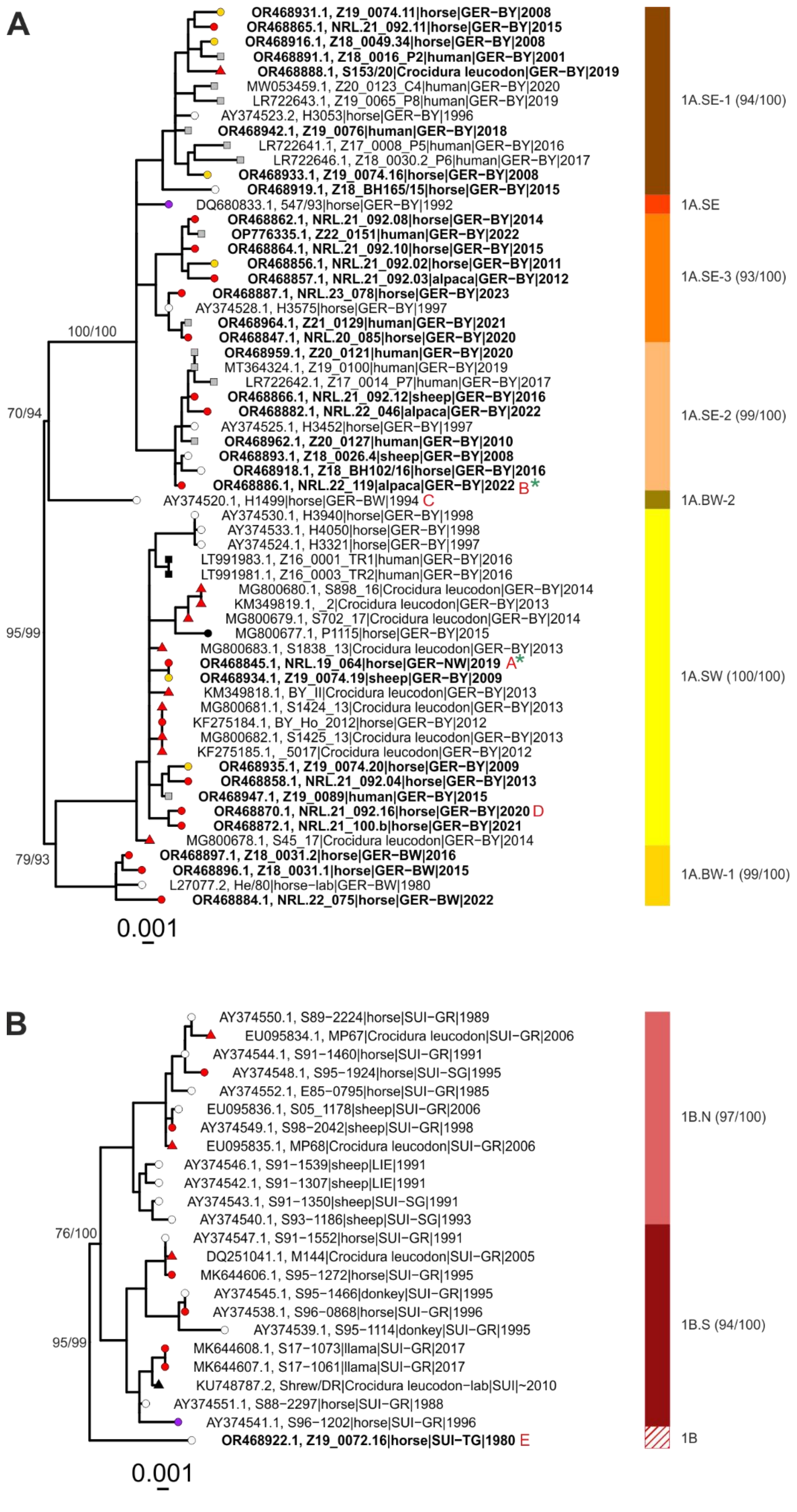
A
OR468931.1, Z19_0074.11|horse|GER-BY|2008
OR468865.1, NRL.21_092.11|horse|GER-BY|2015
OR468916.1, Z18_0049.34|horse|GER-BY|2008
OR468891.1, Z18_0016_P2|human|GER-BY|2001
OR468888.1, S153/20|Crocidura leucodon|GER-BY|2019
MW053459.1, Z20_0123_C4|human|GER-BY|2020
LR722643.1, Z19_0065_P8|human|GER-BY|2019
AY374523.2, H3053|horse|GER-BY|1996
OR468942.1, Z19_0076|human|GER-BY|2018
LR722641.1, Z17_0008_P5|human|GER-BY|2016
LR722646.1, Z18_0030.2_P6|human|GER-BY|2017
OR468933.1, Z19_0074.16|horse|GER-BY|2008
OR468919.1, Z18_BH165/15|horse|GER-BY|2015
DQ680833.1, 547/93|horse|GER-BY|1992
OR468862.1, NRL.21_092.08|horse|GER-BY|2014
OP776335.1, Z22_0151|human|GER-BY|2022
OR468864.1, NRL.21_092.10|horse|GER-BY|2015
OR468856.1, NRL.21_092.02|horse|GER-BY|2011
OR468857.1, NRL.21_092.03|alpaca|GER-BY|2012
OR468887.1, NRL.23_078|horse|GER-BY|2023
AY374528.1, H3575|horse|GER-BY|1997
OR468964.1, Z21_0129|human|GER-BY|2021
OR468847.1, NRL.20_085|horse|GER-BY|2020
OR468959.1, Z20_0121|human|GER-BY|2020
MT364324.1, Z19_0100|human|GER-BY|2019
LR722642.1, Z17_0014_P7|human|GER-BY|2017
OR468866.1, NRL.21_092.12|sheep|GER-BY|2016
OR468882.1, NRL.22_046|alpaca|GER-BY|2022
AY374525.1, H3452|horse|GER-BY|1997
OR468962.1, Z20_0127|human|GER-BY|2010
OR468893.1, Z18_0026.4|sheep|GER-BY|2008
OR468918.1, Z18_BH102/16|horse|GER-BY|2016
OR468886.1, NRL.22_119|alpaca|GER-BY|2022 B*
AY374520.1, H1499|horse|GER-BW|1994 C
AY374530.1, H3940|horse|GER-BY|1998
AY374533.1, H4050|horse|GER-BY|1998
AY374524.1, H3321|horse|GER-BY|1997
LT991983.1, Z16_0001_TR1|human|GER-BY|2016
LT991981.1, Z16_0003_TR2|human|GER-BY|2016
MG800680.1, S898_16|Crocidura leucodon|GER-BY|2014
KM349819.1, _2|Crocidura leucodon|GER-BY|2013
MG800679.1, S702_17|Crocidura leucodon|GER-BY|2014
MG800677.1, P1115|horse|GER-BY|2015
MG800683.1, S1838_13|Crocidura leucodon|GER-BY|2013
OR468845.1, NRL.19_064|horse|GER-NW|2019 A*
OR468934.1, Z19_0074.19|sheep|GER-BY|2009
KM349818.1, BY_II|Crocidura leucodon|GER-BY|2013
MG800681.1, S1424_13|Crocidura leucodon|GER-BY|2013
KF275184.1, BY_Ho_2012|horse|GER-BY|2012
MG800682.1, S1425_13|Crocidura leucodon|GER-BY|2013
KF275185.1, _5017|Crocidura leucodon|GER-BY|2012
OR468935.1, Z19_0074.20|horse|GER-BY|2009
OR468858.1, NRL.21_092.04|horse|GER-BY|2013
OR468947.1, Z19_0089|human|GER-BY|2015
OR468870.1, NRL.21_092.16|horse|GER-BY|2020 D
OR468872.1, NRL.21_100.b|horse|GER-BY|2021
MG800678.1, S45_17|Crocidura leucodon|GER-BY|2014
OR468897.1, Z18_0031.2|horse|GER-BW|2016
OR468896.1, Z18_0031.1|horse|GER-BW|2015
L27077.2, He/80|horse-lab|GER-BW|1980
OR468884.1, NRL.22_075|horse|GER-BW|2022
100/100
70/94
95/99
79/93
0.001
1A.SE-1 (94/100)
1A.SE
1A.SE-3 (93/100)
1A.SE-2 (99/100)
1A.BW-2
1A.SW (100/100)
1A.BW-1 (99/100)
B
AY374550.1, S89-2224|horse|SUI-GR|1989
EU095834.1, MP67|Crocidura leucodon|SUI-GR|2006
AY374544.1, S91-1460|horse|SUI-GR|1991
AY374548.1, S95-1924|horse|SUI-SG|1995
AY374552.1, E85-0795|horse|SUI-GR|1985
EU095836.1, S05_1178|sheep|SUI-GR|2006
AY374549.1, S98-2042|sheep|SUI-GR|1998
EU095835.1, MP68|Crocidura leucodon|SUI-GR|2006
AY374546.1, S91-1539|sheep|LIE|1991
AY374542.1, S91-1307|sheep|LIE|1991
AY374543.1, S91-1350|sheep|SUI-SG|1991
AY374540.1, S93-1186|sheep|SUI-SG|1993
AY374547.1, S91-1552|horse|SUI-GR|1991
DQ251041.1, M144|Crocidura leucodon|SUI-GR|2005
MK644606.1, S95-1272|horse|SUI-GR|1995
AY374545.1, S95-1466|donkey|SUI-GR|1995
AY374538.1, S96-0868|horse|SUI-GR|1996
AY374539.1, S95-1114|donkey|SUI-GR|1995
MK644608.1, S17-1073|llama|SUI-GR|2017
MK644607.1, S17-1061|llama|SUI-GR|2017
KU748787.2, Shrew/DR|Crocidura leucodon-lab|SUI|~2010
AY374551.1, S88-2297|horse|SUI-GR|1988
AY374541.1, S96-1202|horse|SUI-GR|1996
OR468922.1, Z19_0072.16|horse|SUI-TG|1980 E
76/100
95/99
0.001
1B.N (97/100)
1B.S (94/100)
1B

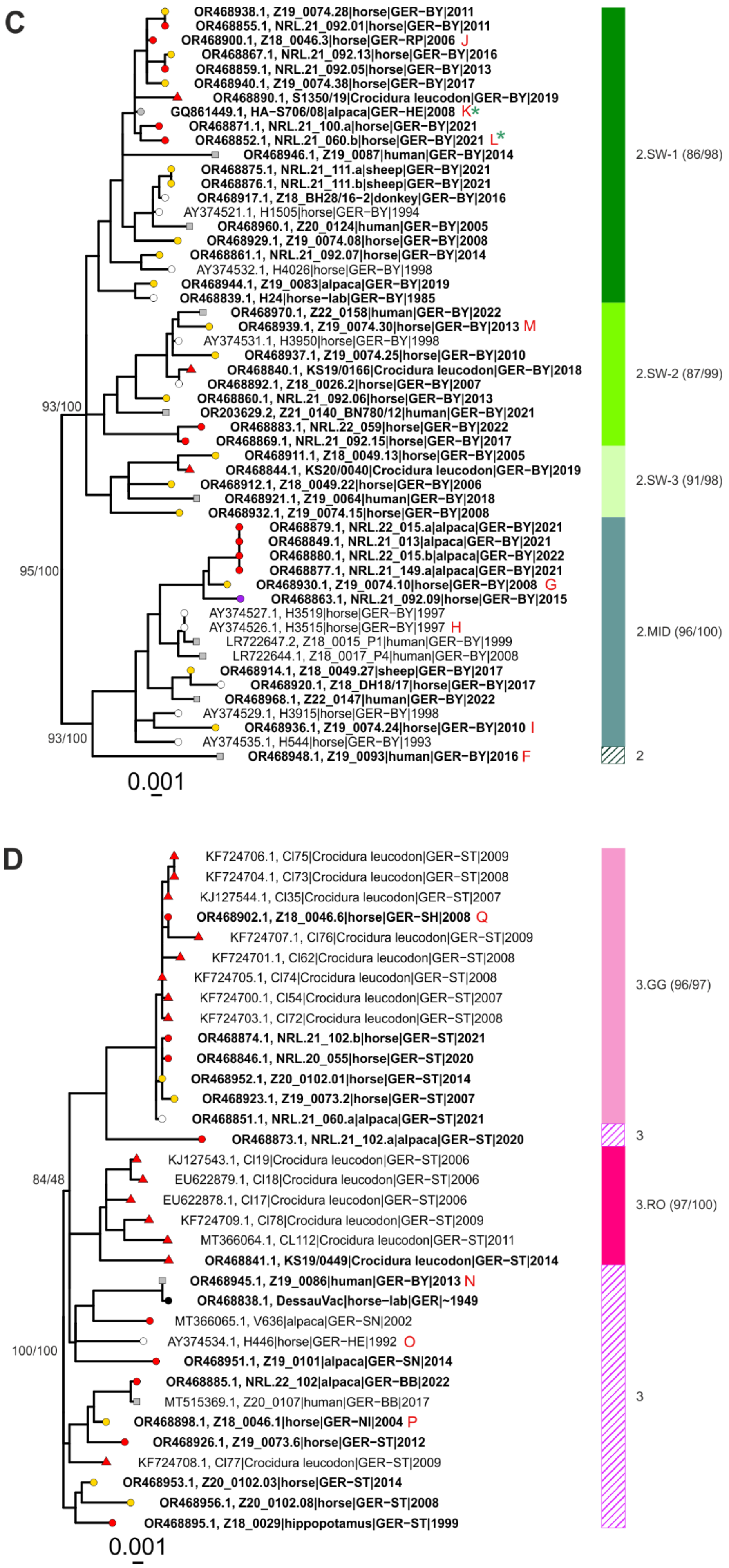
C
OR468938.1, Z19_0074.28|horse|GER-BY|2011
OR468855.1, NRL.21_092.01|horse|GER-BY|2011
OR468900.1, Z18_0046.3|horse|GER-RP|2006 J
OR468867.1, NRL.21_092.13|horse|GER-BY|2016
OR468859.1, NRL.21_092.05|horse|GER-BY|2013
OR468940.1, Z19_0074.38|horse|GER-BY|2017
OR468890.1, S1350/19|Crocidura leucodon|GER-BY|2019
GQ861449.1, HA-S706/08|alpaca|GER-HE|2008 K*
OR468871.1, NRL.21_100.a|horse|GER-BY|2021
OR468852.1, NRL.21_060.b|horse|GER-BY|2021 L*
OR468946.1, Z19_0087|human|GER-BY|2014
OR468875.1, NRL.21_111.a|sheep|GER-BY|2021
OR468876.1, NRL.21_111.b|sheep|GER-BY|2021
OR468917.1, Z18_BH28/16-2|donkey|GER-BY|2016
AY374521.1, H1505|horse|GER-BY|1994
OR468960.1, Z20_0124|human|GER-BY|2005
OR468929.1, Z19_0074.08|horse|GER-BY|2008
OR468861.1, NRL.21_092.07|horse|GER-BY|2014
AY374532.1, H4026|horse|GER-BY|1998
OR468944.1, Z19_0083|alpaca|GER-BY|2019
OR468839.1, H24|horse-lab|GER-BY|1985
OR468970.1, Z22_0158|human|GER-BY|2022
OR468939.1, Z19_0074.30|horse|GER-BY|2013 M
AY374531.1, H3950|horse|GER-BY|1998
OR468937.1, Z19_0074.25|horse|GER-BY|2010
OR468840.1, KS19/0166|Crocidura leucodon|GER-BY|2018
OR468892.1, Z18_0026.2|horse|GER-BY|2007
OR468860.1, NRL.21_092.06|horse|GER-BY|2013
OR203629.2, Z21_0140_BN780/12|human|GER-BY|2021
OR468883.1, NRL.22_059|horse|GER-BY|2022
OR468869.1, NRL.21_092.15|horse|GER-BY|2017
OR468911.1, Z18_0049.13|horse|GER-BY|2005
OR468844.1, KS20/0040|Crocidura leucodon|GER-BY|2019
OR468912.1, Z18_0049.22|horse|GER-BY|2006
OR468921.1, Z19_0064|human|GER-BY|2018
OR468932.1, Z19_0074.15|horse|GER-BY|2008
OR468879.1, NRL.22_015.a|alpaca|GER-BY|2021
OR468849.1, NRL.21_013|alpaca|GER-BY|2021
OR468880.1, NRL.22_015.b|alpaca|GER-BY|2022
OR468877.1, NRL.21_149.a|alpaca|GER-BY|2021
OR468930.1, Z19_0074.10|horse|GER-BY|2008 G
OR468863.1, NRL.21_092.09|horse|GER-BY|2015
AY374527.1, H3519|horse|GER-BY|1997
AY374526.1, H3515|horse|GER-BY|1997 H
LR722647.2, Z18_0015_P1|human|GER-BY|1999
LR722644.1, Z18_0017_P4|human|GER-BY|2008
OR468914.1, Z18_0049.27|sheep|GER-BY|2017
OR468920.1, Z18_DH18/17|horse|GER-BY|2017
OR468968.1, Z22_0147|human|GER-BY|2022
AY374529.1, H3915|horse|GER-BY|1998
OR468936.1, Z19_0074.24|horse|GER-BY|2010 I
AY374535.1, H544|horse|GER-BY|1993
OR468948.1, Z19_0093|human|GER-BY|2016 F
93/100
95/100
93/100
0.001
2.SW-1 (86/98)
2.SW-2 (87/99)
2.SW-3 (91/98)
2.MID (96/100)
2
D
KF724706.1, CI75|Crocidura leucodon|GER-ST|2009
KF724704.1, CI73|Crocidura leucodon|GER-ST|2008
KJ127544.1, CI35|Crocidura leucodon|GER-ST|2007
OR468902.1, Z18_0046.6|horse|GER-SH|2008 Q
KF724707.1, CI76|Crocidura leucodon|GER-ST|2009
KF724701.1, CI62|Crocidura leucodon|GER-ST|2008
KF724705.1, CI74|Crocidura leucodon|GER-ST|2008
KF724700.1, CI54|Crocidura leucodon|GER-ST|2007
KF724703.1, CI72|Crocidura leucodon|GER-ST|2008
OR468874.1, NRL.21_102.b|horse|GER-ST|2021
OR468846.1, NRL.20_055|horse|GER-ST|2020
OR468952.1, Z20_0102.01|horse|GER-ST|2014
OR468923.1, Z19_0073.2|horse|GER-ST|2007
OR468851.1, NRL.21_060.a|alpaca|GER-ST|2021
OR468873.1, NRL.21_102.a|alpaca|GER-ST|2020
KJ127543.1, CI19|Crocidura leucodon|GER-ST|2006
EU622879.1, CI18|Crocidura leucodon|GER-ST|2006
EU622878.1, CI17|Crocidura leucodon|GER-ST|2006
KF724709.1, CI78|Crocidura leucodon|GER-ST|2009
MT366064.1, CL112|Crocidura leucodon|GER-ST|2011
OR468841.1, KS19/0449|Crocidura leucodon|GER-ST|2014
OR468945.1, Z19_0086|human|GER-BY|2013 N
OR468838.1, DessauVac|horse-lab|GER|~1949
MT366065.1, V636|alpaca|GER-SN|2002
AY374534.1, H446|horse|GER-HE|1992 O
OR468951.1, Z19_0101|alpaca|GER-SN|2014
OR468885.1, NRL.22_102|alpaca|GER-BB|2022
MT515369.1, Z20_0107|human|GER-BB|2017
OR468898.1, Z18_0046.1|horse|GER-NI|2004 P
OR468926.1, Z19_0073.6|horse|GER-ST|2012
KF724708.1, CI77|Crocidura leucodon|GER-ST|2009
OR468953.1, Z20_0102.03|horse|GER-ST|2014
OR468956.1, Z20_0102.08|horse|GER-ST|2008
OR468895.1, Z18_0029|hippopotamus|GER-ST|1999
84/48
100/100
0.001
3.GG (96/97)
3
3.RO (97/100)
3

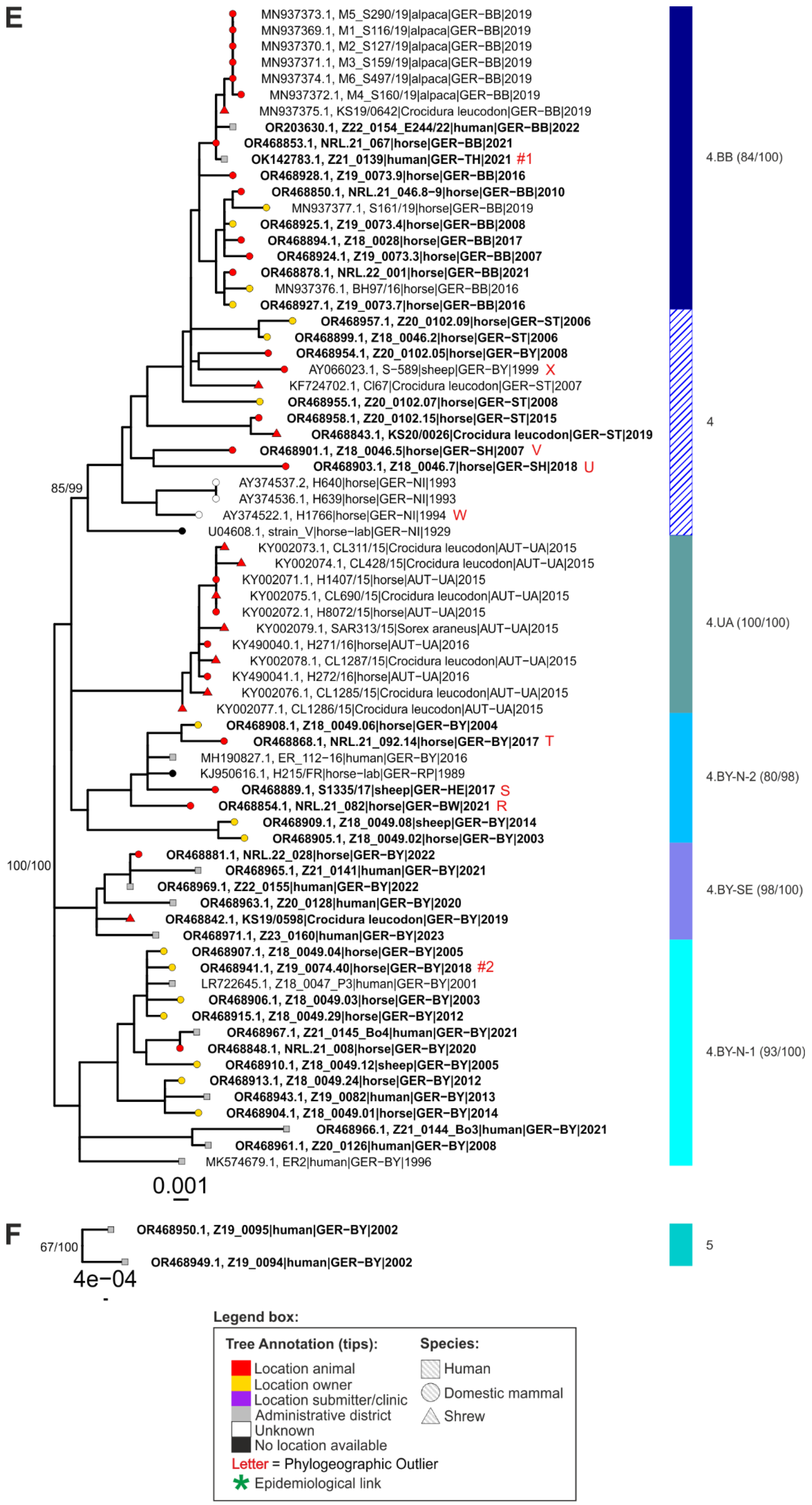
E
MN937373.1, M5_S290/19|alpaca|GER-BB|2019
MN937369.1, M1_S116/19|alpaca|GER-BB|2019
MN937370.1, M2_S127/19|alpaca|GER-BB|2019
MN937371.1, M3_S159/19|alpaca|GER-BB|2019
MN937374.1, M6_S497/19|alpaca|GER-BB|2019
MN937372.1, M4_S160/19|alpaca|GER-BB|2019
MN937375.1, KS19/0642|Crocidura leucodon|GER-BB|2019
OR203630.1, Z22_0154_E244/22|human|GER-BB|2022
OR468853.1, NRL.21_067|horse|GER-BB|2021
OK142783.1, Z21_0139|human|GER-TH|2021 #1
OR468928.1, Z19_0073.9|horse|GER-BB|2016
OR468850.1, NRL.21_046.8-9|horse|GER-BB|2010
MN937377.1, S161/19|horse|GER-BB|2019
OR468925.1, Z19_0073.4|horse|GER-BB|2008
OR468894.1, Z18_0028|horse|GER-BB|2017
OR468924.1, Z19_0073.3|horse|GER-BB|2007
OR468878.1, NRL.22_001|horse|GER-BB|2021
MN937376.1, BH97/16|horse|GER-BB|2016
OR468927.1, Z19_0073.7|horse|GER-BB|2016
OR468957.1, Z20_0102.09|horse|GER-ST|2006
OR468899.1, Z18_0046.2|horse|GER-ST|2006
OR468954.1, Z20_0102.05|horse|GER-BY|2008
AY066023.1, S-589|sheep|GER-BY|1999 X
KF724702.1, Cl67|Crocidura leucodon|GER-ST|2007
OR468955.1, Z20_0102.07|horse|GER-ST|2008
OR468958.1, Z20_0102.15|horse|GER-ST|2015
OR468843.1, KS20/0026|Crocidura leucodon|GER-ST|2019
OR468901.1, Z18_0046.5|horse|GER-SH|2007 V
OR468903.1, Z18_0046.7|horse|GER-SH|2018 U
85/99
AY374537.2, H640|horse|GER-NI|1993
AY374536.1, H639|horse|GER-NI|1993
AY374522.1, H1766|horse|GER-NI|1994 W
U04608.1, strain_V|horse-lab|GER-NI|1929
KY002073.1, CL311/15|Crocidura leucodon|AUT-UA|2015
KY002074.1, CL428/15|Crocidura leucodon|AUT-UA|2015
KY002071.1, H1407/15|horse|AUT-UA|2015
KY002075.1, CL690/15|Crocidura leucodon|AUT-UA|2015
KY002072.1, H8072/15|horse|AUT-UA|2015
KY002079.1, SAR313/15|Sorex araneus|AUT-UA|2015
KY490040.1, H271/16|horse|AUT-UA|2016
KY002078.1, CL1287/15|Crocidura leucodon|AUT-UA|2015
KY490041.1, H272/16|horse|AUT-UA|2016
KY002076.1, CL1285/15|Crocidura leucodon|AUT-UA|2015
KY002077.1, CL1286/15|Crocidura leucodon|AUT-UA|2015
OR468908.1, Z18_0049.06|horse|GER-BY|2004
OR468868.1, NRL.21_092.14|horse|GER-BY|2017 T
MH190827.1, ER_112-16|human|GER-BY|2016
KJ950616.1, H215/FR|horse-lab|GER-RP|1989
OR468889.1, S1335/17|sheep|GER-HE|2017 S
OR468854.1, NRL.21_082|horse|GER-BW|2021 R
OR468909.1, Z18_0049.08|sheep|GER-BY|2014
OR468905.1, Z18_0049.02|horse|GER-BY|2003
100/100
OR468881.1, NRL.22_028|horse|GER-BY|2022
OR468965.1, Z21_0141|human|GER-BY|2021
OR468969.1, Z22_0155|human|GER-BY|2022
OR468963.1, Z20_0128|human|GER-BY|2020
OR468842.1, KS19/0598|Crocidura leucodon|GER-BY|2019
OR468971.1, Z23_0160|human|GER-BY|2023
OR468907.1, Z18_0049.04|horse|GER-BY|2005
OR468941.1, Z19_0074.40|horse|GER-BY|2018 #2
LR722645.1, Z18_0047_P3|human|GER-BY|2001
OR468906.1, Z18_0049.03|horse|GER-BY|2003
OR468915.1, Z18_0049.29|horse|GER-BY|2012
OR468967.1, Z21_0145_Bo4|human|GER-BY|2021
OR468848.1, NRL.21_008|horse|GER-BY|2020
OR468910.1, Z18_0049.12|sheep|GER-BY|2005
OR468913.1, Z18_0049.24|horse|GER-BY|2012
OR468943.1, Z19_0082|human|GER-BY|2013
OR468904.1, Z18_0049.01|horse|GER-BY|2014
OR468966.1, Z21_0144_Bo3|human|GER-BY|2021
OR468961.1, Z20_0126|human|GER-BY|2008
MK574679.1, ER2|human|GER-BY|1996
0.001
4.BB (84/100)
4
4.UA (100/100)
4.BY-N-2 (80/98)
4.BY-SE (98/100)
4.BY-N-1 (93/100)
F
67/100
OR468950.1, Z19_0095|human|GER-BY|2002
OR468949.1, Z19_0094|human|GER-BY|2002
4e−04
5
Legend box:
Tree Annotation (tips):
Location animal
Location owner
Location submitter/clinic
Administrative district
Unknown
No location available
Letter = Phylogeographic Outlier
Epidemiological link
Species:
Human
Domestic mammal
Shrew

Supplementary Figure 5. Phylogenetic analysis of partial BoDV-1 sequences covering the N-X/P genes. A maximum likelihood (model GTR+F+I+G4) tree was calculated for 246 partial BoDV-1 sequences (1,824 nucleotides) of human and animal origin covering the complete N, X and P genes (genome positions 54 to 1877). **A) to F)** Subtrees of this tree are presented individually for spatial distribution of subclusters 1A **(A)** and 1B **(B)** and clusters 2 **(C)**, 3 **(D)**, 4 **(E)** and 5 **(F)**. Sequence BoDV-2 No/98 (AJ311524; not displayed) was used to root the tree. Statistical support is shown for main branches (including clusters, subclusters, and subclades), using the format "SH-aLRT/ultrafast bootstrap". Clusters 2 to 5 and subclusters 1A and 1B are indicated by coloured branches. Subclades are indicated by coloured bars and corresponding text labels, with statistical support of subclades shown in brackets. Coloured symbols at tips represent the reliability of the available geographic information (see legend box). Red labels represent phylogeographic outliers (capital letters; Supplementary Table 3) or additional cases with potentially aberrant infection site (#1 and #2; Supplementary Table 4). Asterisks indicate known epidemiologic links into the dispersal area of the respective subclade. Germany (GER): BB = Brandenburg, BY = Bavaria, BW = Baden-Wuerttemberg, HE = Hesse, NI = Lower Saxony, NW = North Rhine-Westphalia, RP = Rhineland-Palatinate; SH = Schleswig-Holstein, SN = Saxony, ST = Saxony-Anhalt, TH = Thuringia; Switzerland (SUI): GR = Grisons, SG = St. Gall, TG = Thurgau; Austria (AUT): UA = Upper Austria; Liechtenstein (LIE). Subclade designations: GG = Güterglück, MID = Middle, N = North, S = South, RO = Rosslau, SE = Southeast, SW = Southwest.

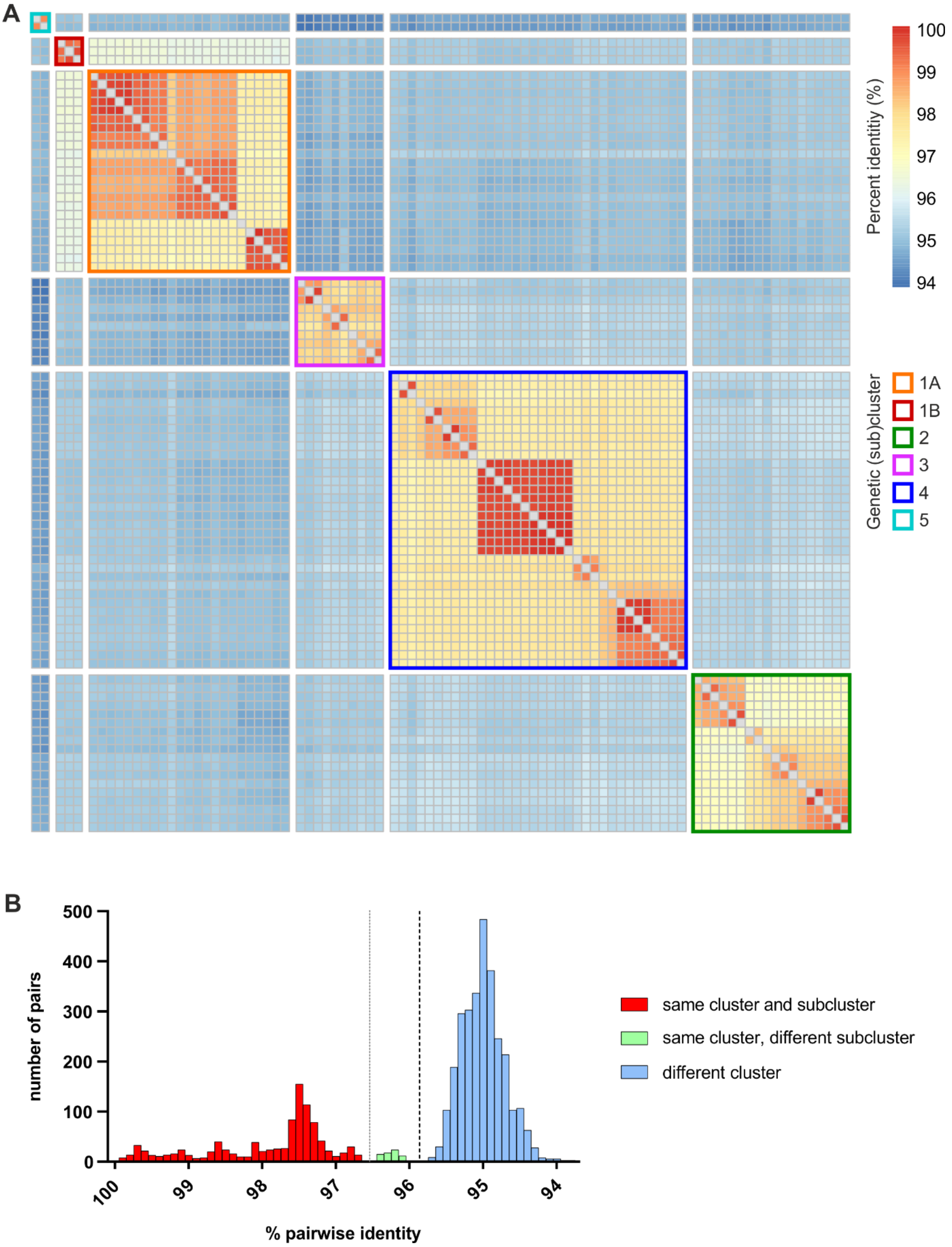

Supplementary Figure 6. Cluster demarcation based on pairwise nucleotide sequence identities. A) Heatmap of nucleotide (nt) sequence identities of all 90 complete coding BoDV-1 genomes inferred from the nt sequence alignment used for the maximum likelihood phylogenetic tree (Figure 2). **B)** Frequencies of pairwise nt sequence identities within and between BoDV-1 clusters and subclusters. Broken lines represent cluster demarcation (95.8%) and demarcation of subclusters 1A and 1B (96.6%).

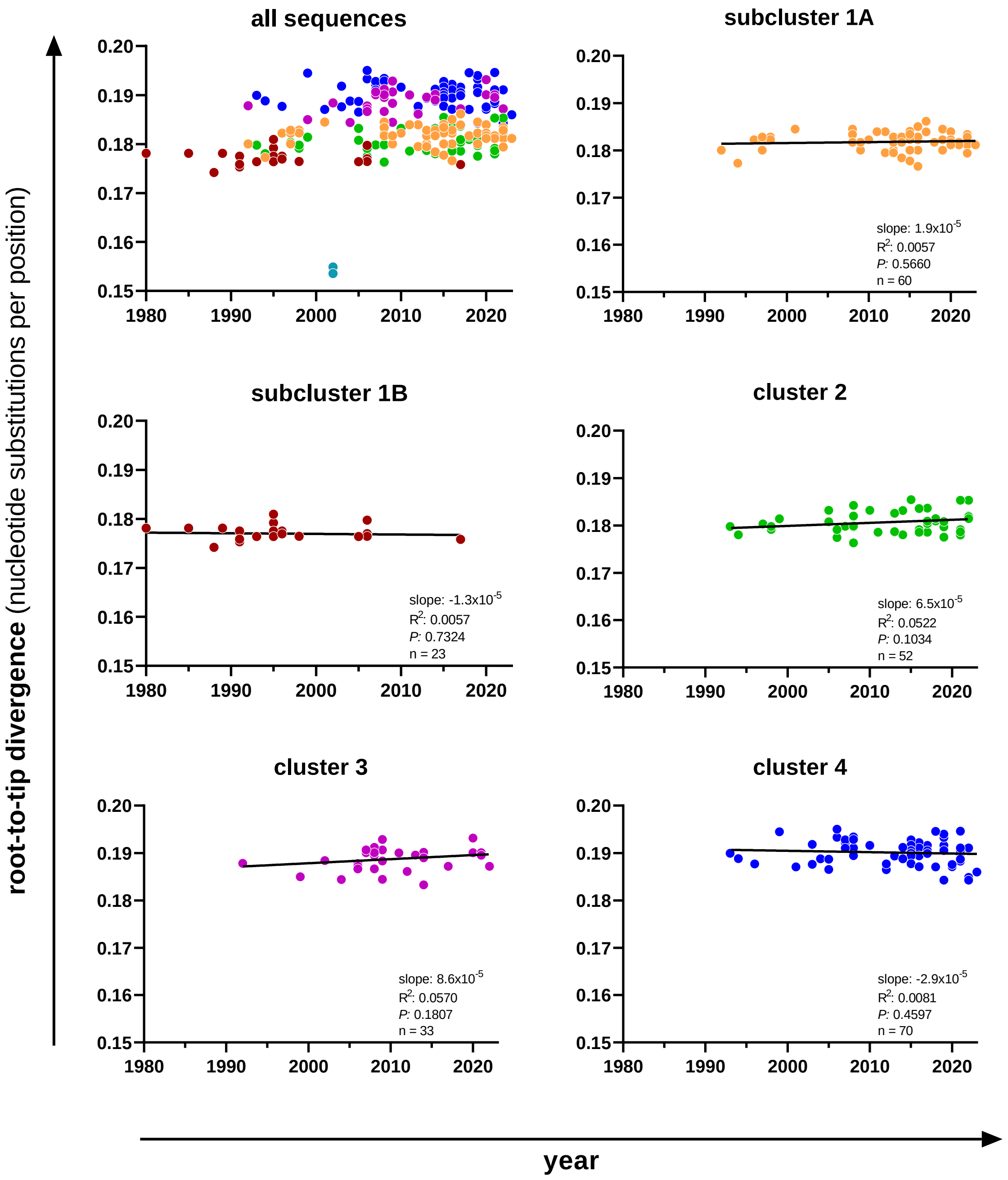

Supplementary Figure 7. Linear regression analyses of genetic divergence against year of sampling. Root-to-tip distances were inferred for 240 sequences from the maximum likelihood (ML) tree of N-X/P nucleotide sequences (Figure 3A; Supplementary Figure 5). Laboratory strains and sequences without available year of sampling were excluded from the analysis. Black lines represent simple linear regression curves. Slope, goodness of fit (R^2) and statistical significance of the slope being different from 0 ($P<0.05$; F test) are provided.

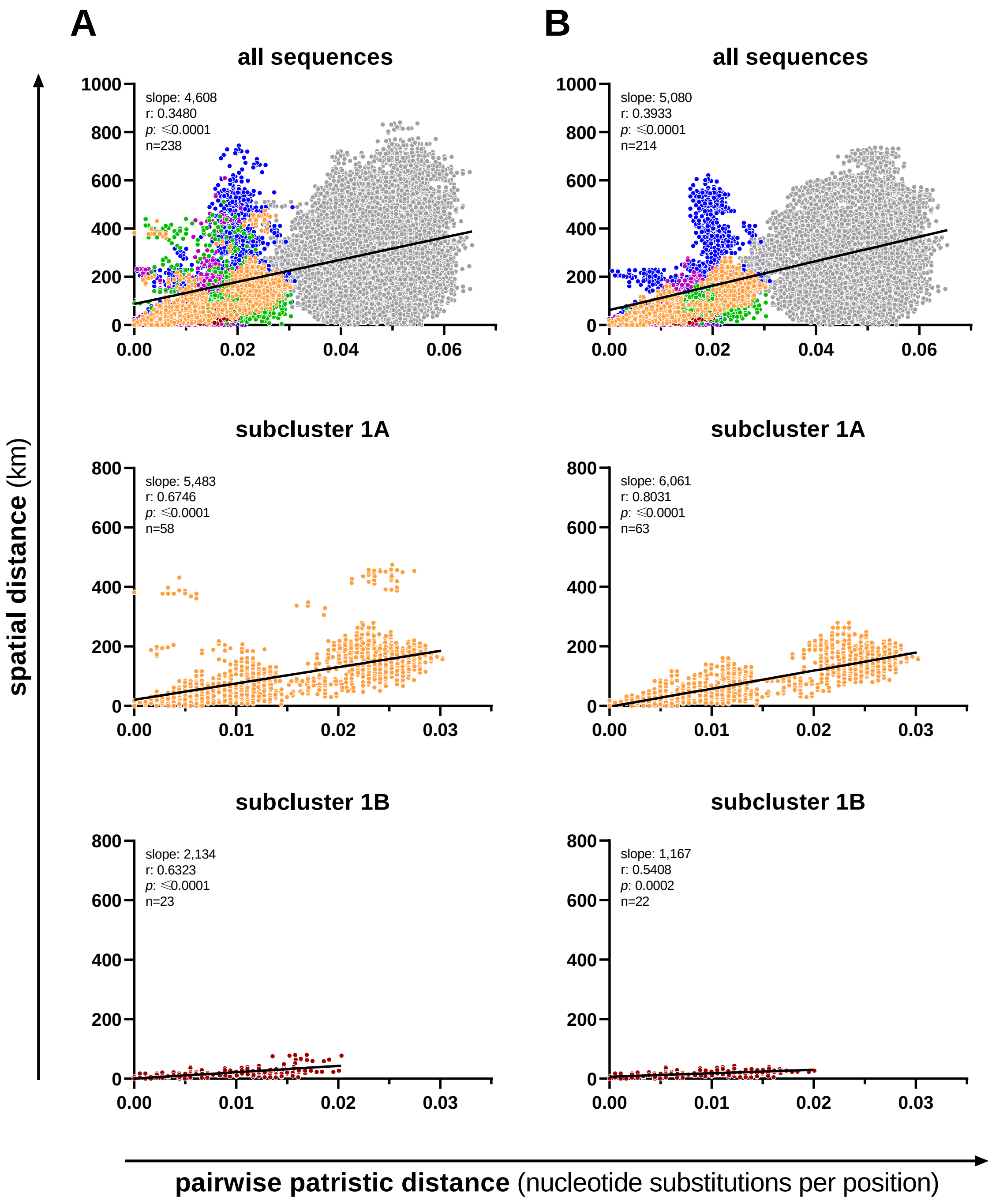

(continues on next page)

(continued from previous page)

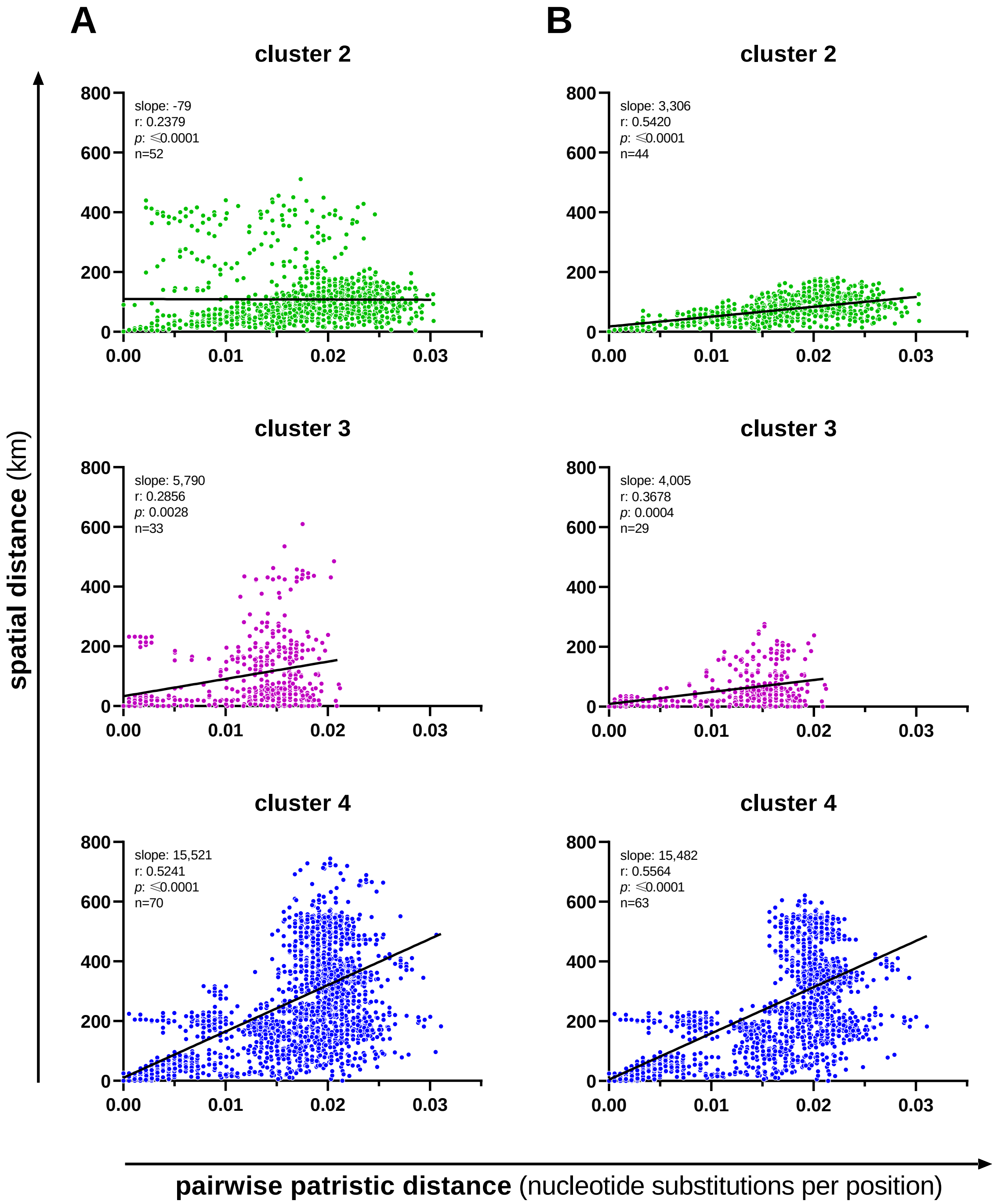

Supplementary Figure 8. Analysis of pairwise spatial versus phylogenetic distances. Pairwise patristic distances were inferred from the maximum likelihood (ML) tree of N-X/P nucleotide sequences (Figure 3A; Supplementary Figure 5) and plotted against pairwise spatial distances. **A)** Results of all 238 N-X/P nucleotide sequences with available locations. **B)** Results of 214 N-X/P sequences after removal of the 24 sequences that were categorized as phylogeographic outliers using the following criteria: existence of no other BoDV-1 N-X/P sequence with ≥98.6% nucleotide sequence identity within a distance of ≤37.9 km. Black lines represent linear regression curves. A Mantel test was performed and the resulting correlation coefficient (r) and *p*-value are shown.

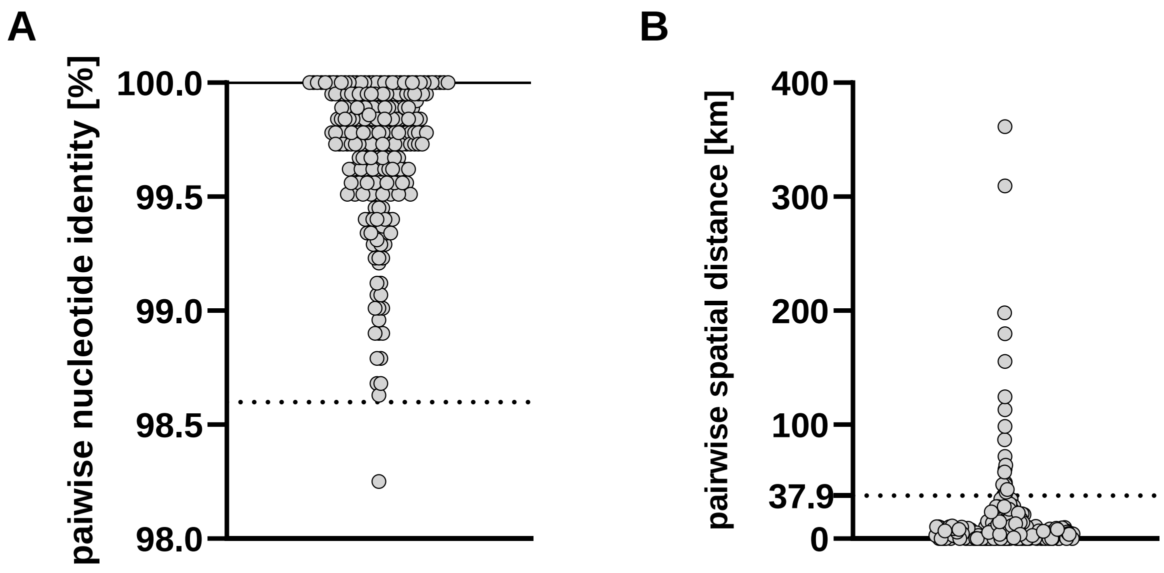

Supplementary Figure 9. Criteria for the determination of phylogeographic outliers. A) Maximal pairwise nucleotide (nt) sequence identities for each of 238 N-X/P sequences with available location. All but one sequence possessed at least one relative with ≥98.6% nt sequence identity (dotted line) **B)** Minimal pairwise spatial distances for each case to all other cases with at least 98.6% N-X/P nt sequence identity. The dotted line represents the 90th percentile of the dataset (37.9 km).

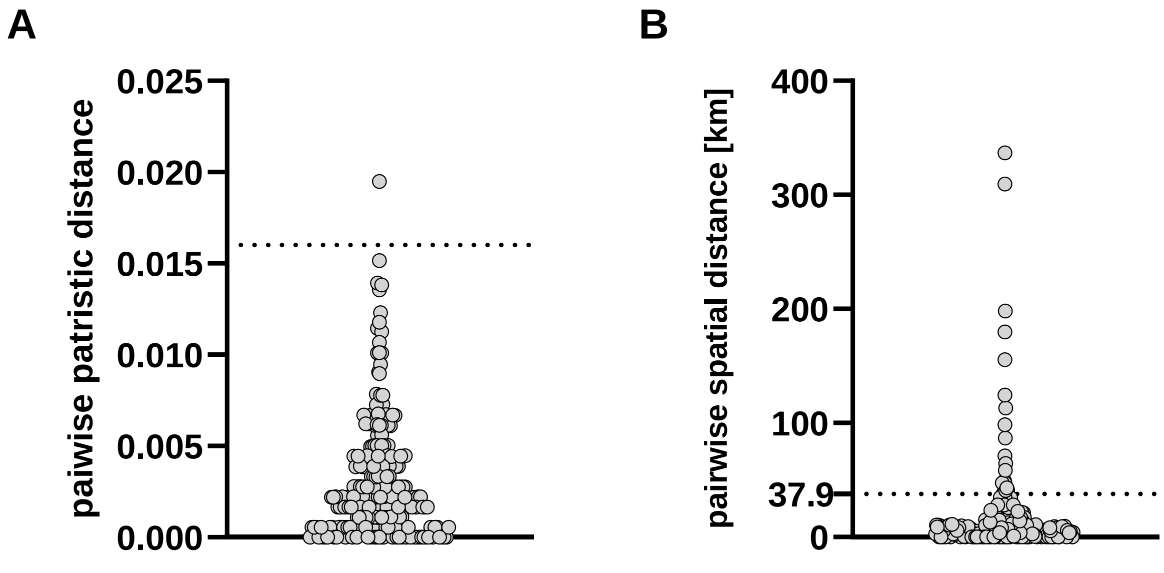

Supplementary Figure 10. Determination of phylogeographic outliers using patristic distances. A) Minimal pairwise patristic distance inferred from the maximum likelihood (ML) tree of N-X/P nucleotide sequences (Figure 3A; Supplementary Figure 5) for each of 238 N-X/P sequences with available location. All but one sequence possessed at least one relative with a patristic distance of ≤0.016 (dotted line) **B)** Minimal pairwise spatial distances for each case to all other cases with a patristic distance of ≤0.016. The dotted line represents the 90th percentile of the dataset (37.9 km).
Using this comparative approach, the same 24 phylogeographic outliers were determined as identified by using nucleotide sequence identities (Supplementary Table 3).

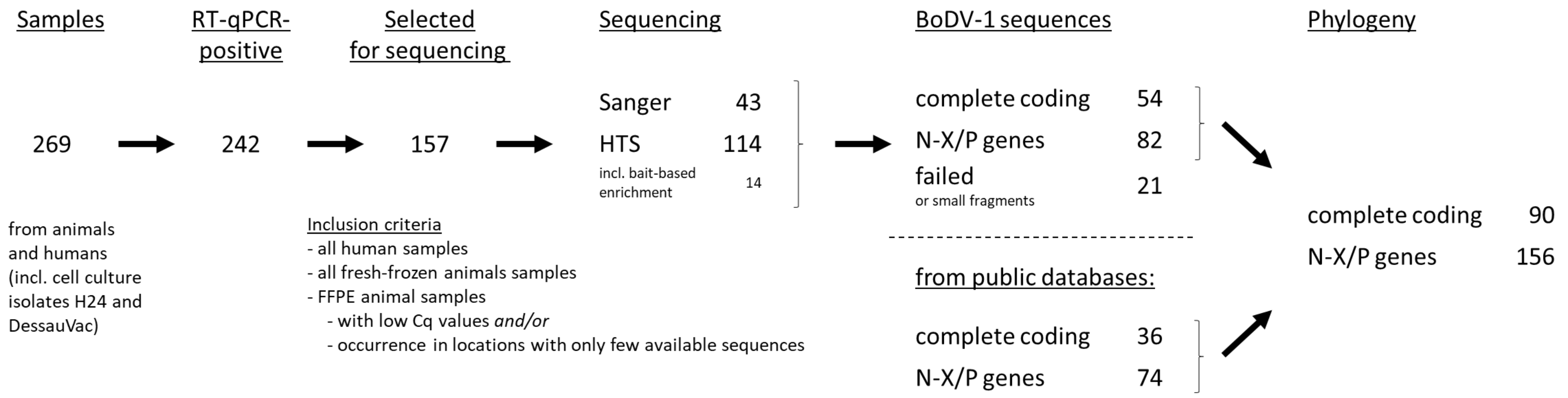

Supplementary Figure 11. Schematic summary of the analysis workflow. Samples for this study were obtained from human and veterinary diagnostic and pathology laboratories. Samples were screened by BoDV-1-specific RT-qPCR to confirm BoDV-1 infection. BoDV-1-positive samples were selected for sequencing based on the inclusion criteria summarized in the figure. Fresh-frozen samples were preferentially analysed using Sanger sequencing of conventional RT-PCR amplicons covering the N-X/P genes (1,824 nt). High throughput sequencing (HTS) was performed for FFPE samples as well as for selected fresh-frozen samples from which generation of a complete BoDV-1 genome was intended. BoDV-1 target enrichment in cDNA libraries was attempted to increase sequencing depth when untargeted HTS did not provide sufficient sequence quality. HTS-derived sequences covered either the complete coding sequence of the BoDV-1 genome (8,769 nt) or at least the N-X/P genes. Short gaps in HTS-derived sequences were closed by Sanger sequencing of RT-PCR amplicons. Sequence data consicting only of short fragments was not further analysed. For phylogenetic analysis, the sequences generated in this study were combined with previously published BoDV-1 sequences derived from public databases. A detailed summary of sample and sequences numbers is provided in Table 1. All BoDV-1 sequences generated during this study (OR203629, OR203630, OR468838 to OR468971) are summarized in Supplementary Data 1in a separate file.

Supplementary Table 1. Domestic mammals included in this study (incl. zoo animals)

Animal	Species	Analysed in this study	Confirmed by RT-qPCR in this study	Confirmed cases incl. published sequences [a]
horse	*Equus caballus*	178	162	198
sheep	*Ovis gmelini aries*	31	26	33
alpaca	*Vicugna pacos*	16	16	24
pygmy hippopotamus	*Choeropsis liberiensis*	2	1	1
cattle	*Bos taurus*	2	0	0
donkey	*Equus asinus*	1	1	3
dog	*Canis lupus familiaris*	1	1	1
llama	*Lama glama*	-	-	2

[a] Only sequences from public databases covering at least the complete N, X and P genes were included.

Supplementary Table 2. Primers and probes used for BoDV-1 RNA detection, housekeeping gene amplification and Sanger sequencing.

Assay	Primer/Probe name	Sequence (5' to 3')	Reference
BoDV-1 mix 1	BoDV-1_1258+	TAGTYAGGAGGCTCAATGGCA	[1]
	BoDV-1_1316_FAM	FAM-AAGAAGATCCCCAGACACTACGACG-BHQ1	[1]
	BoDV-1_1419-	GTCCYTCAGGAGCTGGTC	[1]
BoDV-1 mix 6	BoDV-1_2231+	CAATYAATGCAGCYTTCAATGTCTT	[1]
	BoDV-1_2285as_FAM	FAM-CCARCACCAATGTTCCGAAGCCG-BHQ1	[1]
	BoDV-1_2305-	GAATGTCYGGGCCGAGAG	[1]
beta actin mix 2	ACT_F_1005-1029	CAGCACAATGAAGATCAAGATCATC	[2]
	ACT_P_1081-1105_HEX	HEX-TCGCTGTCCACCTTCCAGCAGATGT-BHQ1	[2]
	ACT_R_1135-1114	CGGACTCATCGTACTCCTGCTT	[2]
eGFP mix 1	EGFP-1-F	GACCACTACCAGCAGAACAC	[3]
	EGFP-Probe1_HEX	HEX-AGCACCCAGTCCGCCCTGAGCA-BHQ1	[3]
	EGFP-2-R	GAACTCCAGCAGGACCATG	[3]
BoDV-1 amplification for sequencing	PaBV-2_1+	TGTTGCGGTAACAACCAAC	[4]
	BoDV-1_1161-	TTAGACCAGTCACACCTATC	[5]
	BoDV-1_1068+	GTATAGGCGCCGCGAGATAT	[5]
	BoDV-1_2311-	AAGATCGAATGTCTGGGCCG	[5]
sequencing primers	BoDV-1_523+	GCAGGAGCCGARCAGATCAAG	[5]
	BoDV-1_656-	GGTTGGCCGTTAATCCAATC	[5]
	BoDV-1_1621+	GAAACCATCCAGACAGCTCAG	[5]
	BoDV-1_1816-	GAGGTGCAGGATGGGAGGG	[5]

ACT: actin; BoDV-1: Borna disease virus 1; eGFP: enhanced green fluorescent protein; FAM: 6-Carboxyfluorescein; HEX: hexachlorfluorescein; PaBV-2: parrot bornavirus 2

Supplementary Table 3. Phylogeographic outliers identified during this study.

Outlier ID	*Accession no.*	*Animal ID*	*Host*	*Country & federal state*	*Year*	*Cluster or sub-cluster& subclade*	*Location status [b]*	*Known links to dispersal area of the respective subclade*		*Distance to closest phylogenetic relative [a]*					
										Accession no.	*Host*	*Country & federal state*	*Year*	*nt identity (%)*	*Distance (km)*
A	OR468845	NRL.19_064	horse	GER-NW	2019	1A.SW	1	yes	bought from south-western GER-BY approximately two months before death	OR468934	sheep	GER-BY	2009	100	382
B	OR468886	NRL.22_119	alpaca	GER-BY	2022	1A.SE-2	1	yes	bought from south-eastern GER-BY eight months before death; chronically atactic at the time of arrival	OR468893	sheep	GER-BY	2008	99.8	188
C	AY374520	H1499	horse	GER-BW	1994	1A.BW-2	5	n.a. [c]	-	OR468897	horse	GER-BW	2016	98.5	71
D	OR468870	NRL.21_092.16	horse	GER-BY	2020	1A.SW	1	n.a.	-	OR468897	horse	GER-BY	2021	99.8	49
E	OR468922	Z19_0072.16	horse	SUI-TG	1980	1B	5	n.a.	-	AY374551	horse	SUI-GR	1988	98.7	76
F	OR468948	Z18_0093	human	GER-BY	2016	2	4	n.a.	-	AY374535	horse	GER-BY	1993	98.1	17
G	OR468930	Z19_0074.10	horse	GER-BY	2008	2.MID	2	n.a.	-	OR468849	alpaca	GER-BY	2021	99.6	53
H	AY374526	H3515	horse	GER-BY	1997	2.MID	5	n.a.	-	AY374527	horse	GER-BY	1997	100	90
I	OR468936	Z19_0074.24	horse	GER-BY	2010	2.MID	2	n.a.	-	AY374529	horse	GER-BY	1998	99.2	56
J	OR468900	Z18_0046.3	horse	GER-NW	2006	2.SW-1	1	no	born in GER-HE, later transferred to GER-RP and GER-NW; no links to endemic regions reported	OR468938	horse	GER-BY	2011	99.8	413
K	GQ861449	HA-S706/08	alpaca	GER-HE	2008	2.SW-1	4	yes	introduced to the herd shortly before onset of disease; origin southwestern Bavaria [6]	OR468900<br>OR468940	horse<br>horse	GER-NW<br>GER-BY	2006<br>2017	99.8<br>99.7	198<br>395
L	OR468852	NRL_21.060.b	horse	GER-BY	2021	2.SW-1	1	yes	bought from a horse trader in GER-BW two weeks before death; trader reported to have bought the horse from GER-BY several weeks before (without further specification); reported to be born in Hungary	GQ861449<br>OR468900<br>OR468855	alpaca<br>horse<br>horse	GER-HE<br>GER-NW<br>GER-BY	2008<br>2006<br>2011	99.7<br>99.6<br>99.5	385<br>219<br>270
M	OR468939	Z19_0074.30	horse	GER-BY	2013	2.SW-2	2	n.a.	-	AY374531	horse	GER-BY	1998	99.6	140
N	OR468945	Z19_0086	human	GER-BY	2013	3	4	n.a.	-	OR468838<br>MT366065	vaccine<br>alpaca	GER<br>GER-SN	~1949<br>2002	99.9<br>98.9	n.a.<br>366
O	AY374534	H446	horse	GER-HE	1992	3	5	n.a.	-	OR468953	horse	GER-ST	2014	99.0	196
P	OR468898	Z18_0046.1	horse	GER-NI	2004	3	2	no	originated from ~50 km further south in GER-NI; no reported link to known endemic regions	OR468926	horse	GER-ST	2012	99.5	153
Q	OR468902	Z18_0046.6	horse	GER-SH	2008	3.GG	1	no	bought 4 years before death; origin unknown	KF724705	shrew [e]	GER-ST	2008	100	232

continued on next page

continued from previous page

Outlier ID	*Accession no.*	*Animal ID*	*Host*	*Country & federal state*	*Year*	*Cluster or sub-cluster & subclade*	*Location status* [b]	*Known links to dispersal area of the respective subclade*		**Distance to closest phylogenetic relative** [a]					
										Accession no.	*Host*	*Country & federal state*	*Year*	*nt identity (%)*	*Distance (km)*
R	OR468854	NRL.21_082	horse	GER-BW	2021	4.BY-N-2	1	n.a.	-	MH190827	human	GER-BY	2016	99.3	74
S	OR468889	S1335/17	sheep	GER-HE	2017	4.BY-N-2	1	n.a.	singular case in the herd; no information on the origin of the individual	MH190827	human	GER-BY	2016	99.4	140
T	OR468868	NRL.21_092.14	horse	GER-BY	2017	4.BY-N-2	1	n.a.	-	OR468908	horse	GER-BY	2004	99.6	49
U	OR468903	Z18_0046.7	horse	GER-SH	2018	4	1	no	born in the holding and reported to have never left it	OR468901	horse	GER-SH	2007	98.7	65
V	OR468901	Z18_0046.5	horse	GER-SH	2007	4	1	n.a.	-	OR468853	horse	GER-BB	2021	98.8	195
W	AY374522	H1766	horse	GER-NI	1994	4	5	n.a.	-	AY374536	horse	GER-NI	1993	99.3	99
X	AY066023	S-589	sheep	GER-BY	1999	4	1	no	from a herd with several reported BoDV-1 infections prior to this case; animal was part of the herd since at least one year before death [7]	OR468853	horse	GER-BB	2021	99.2	317

[a] Additional hits are included if no location was available for the closest relative or the closest relative was itself regarded as a phylogeographic outlier.

[b] Location status: 1 = location of animal, 2 = location of owner, 4 = county, 5 = unknown

[c] n.a. = no information available

[d] *Crocidura leucodon*

Germany (GER): BB = Brandenburg, BY = Bavaria, BW = Baden-Wuerttemberg, HE = Hesse, NI = Lower Saxony, NW = North Rhine-Westphalia, RP = Rhineland-Palatinate; SH = Schleswig-Holstein, SN = Saxony, ST = Saxony-Anhalt, Switzerland (SUI): GR = Grisons, TG = Thurgau. Subclade designations: GG = Güterglück, MID = Middle, N = North, SE = Southeast, SW = Southwest.

Supplementary Table 4. Additional cases suspected to possess aberrant infection sources.

These two cases do not match the criteria of phylogeographic outliers, since they are located at ≤37.9 km to at least one case with ≥98.6% BoDV-1 nucleotide (nt) sequence identity. They are nevertheless suspected to have aberrant infection sources due to possessing markedly higher sequence identities (≥99.7%) to sequences at great distances. They are labelled and listed solely for the information of the reader, but were not treated as phylogeographic outliers in any subsequent analysis.

Outlier ID	*Accession no.*	*Animal ID*	*Host*	*Country & federal state*	*Year*	*Cluster or sub-cluster& subclade*	*Lo-cation status* [a]	*Known links to dispersal area of the respective subclade*		**Distance to closest phylogenetic relative as compared to the genetically closest case located at a distance of ≤37.9 km and the spatially closest case with ≥98.6% BoDV-1 nt sequence identity**					
										Accession no.	*Host*	*Country & federal state*	Year	*nt identity (%)*	*Distance (km)*
#1	OK142783	Z21_00139	human	GER-TH	2021	4.BB	4	no	reported to not have travelled outside GER-TH for 8 months [8]	OR468853	horse	GER-BB	2021	99.9	224
										KF724702	shrew [c]	GER-ST	2007	99.3	37
										OR468955	horse	GER-ST	2008	99.2	26
#2	OR468941	Z19_0074.40	horse	GER-BY	2018	4.BY-N-1	2	n.a. [b]	-	OR468907	horse	GER-BY	2005	99.7	199
										OR468881	horse	GER-BY	2022	98.7	30
										OR468969	human	GER-BY	2022	98.7	24

[a] Location status: 2 = location of owner, 4 = county

[b] n.a. = no information available

[c] *Crocidura leucodon*Germany (GER): BB = Brandenburg, BY = Bavaria, ST = Saxony-Anhalt, TH = Thuringia. Subclade designations: N = North.

References

1. Schlottau K*, et al.* Fatal encephalitic Borna disease virus 1 in solid-organ transplant recipients. *N Engl J Med* **379**, 1377-1379 (2018).

2. Toussaint JF, Sailleau C, Breard E, Zientara S, De Clercq K. Bluetongue virus detection by two real-time RT-qPCRs targeting two different genomic segments. *J Virol Methods* **140**, 115-123 (2007).

3. Hoffmann B, Depner K, Schirrmeier H, Beer M. A universal heterologous internal control system for duplex real-time RT-PCR assays used in a detection system for pestiviruses. *J Virol Methods* **136**, 200-209 (2006).

4. Rubbenstroth D, Schmidt V, Rinder M, Legler M, Twietmeyer S, Schwemmer P, Corman VM. Phylogenetic analysis supports horizontal transmission as a driving force of the spread of avian bornaviruses. *PLoS One* **11**, e0160936 (2016).

5. Schulze V*, et al.* Borna disease outbreak with high mortality in an alpaca herd in a previously unreported endemic area in Germany. *Transbound Emerg Dis* **67**, 2093-2107 (2020).

6. Jacobsen B*, et al.* Borna disease in an adult alpaca stallion (*Lama pacos*). *J Comp Pathol* **143**, 203-208 (2010).

7. Vahlenkamp TW, Konrath A, Weber M, Muller H. Persistence of Borna disease virus in naturally infected sheep. *J Virol* **76**, 9735-9743 (2002).

8. Frank C*, et al.* Human Borna disease virus 1 (BoDV-1) encephalitis cases in the north and east of Germany. *Emerg Microbes Infect* **11**, 6-13 (2022).
