# Supplementary material for: Lethal Borna disease virus 1 infections of humans and animals – in-depth molecular epidemiology and phylogeography
Source: Nat Commun. 2024 Sep 10;15:7908. doi: 10.1038/s41467-024-52192-x (PMC11387626; doi:10.1038/s41467-024-52192-x)
Supplement: Supplementary file 3 — Description of Additional Supplementary Files [file 41467_2024_52192_MOESM3_ESM.pdf]

Description of Additional Supplementary Files

File Name: Supplementary Data 1
Description: Accession numbers, sequencing procedure and metadata on all BoDV-1 sequences newly generated during this study.

File Name: Supplementary Data 2
Description: Phylogenetic tree underlying Figure 2 (complete coding genomes) and Figure 4/Supplementary Figure 5 (N-X/P sequences) in Newick format.
