# Supplementary material for: Lethal Borna disease virus 1 infections of humans and animals – in-depth molecular epidemiology and phylogeography
Source: Nat Commun. 2024 Sep 10;15:7908. doi: 10.1038/s41467-024-52192-x (PMC11387626; doi:10.1038/s41467-024-52192-x)
Supplement: Supplementary file 6 — Reporting Summary [file 41467_2024_52192_MOESM6_ESM.pdf]

nature portfolio

Corresponding author(s): Dennis Rubbenstroth

Last updated by author(s): Jul 30, 2024

Reporting Summary

Nature Portfolio wishes to improve the reproducibility of the work that we publish. This form provides structure for consistency and transparency in reporting. For further information on Nature Portfolio policies, see our Editorial Policies and the Editorial Policy Checklist.

Statistics

For all statistical analyses, confirm that the following items are present in the figure legend, table legend, main text, or Methods section.

n/a	Confirmed	
☐	☒	The exact sample size (*n*) for each experimental group/condition, given as a discrete number and unit of measurement
☐	☒	A statement on whether measurements were taken from distinct samples or whether the same sample was measured repeatedly
☐	☒	The statistical test(s) used AND whether they are one- or two-sided<br>*Only common tests should be described solely by name; describe more complex techniques in the Methods section.*
☒	☐	A description of all covariates tested
☒	☐	A description of any assumptions or corrections, such as tests of normality and adjustment for multiple comparisons
☐	☒	A full description of the statistical parameters including central tendency (e.g. means) or other basic estimates (e.g. regression coefficient) AND variation (e.g. standard deviation) or associated estimates of uncertainty (e.g. confidence intervals)
☐	☒	For null hypothesis testing, the test statistic (e.g. *F*, *t*, *r*) with confidence intervals, effect sizes, degrees of freedom and *P* value noted<br>*Give P values as exact values whenever suitable.*
☒	☐	For Bayesian analysis, information on the choice of priors and Markov chain Monte Carlo settings
☒	☐	For hierarchical and complex designs, identification of the appropriate level for tests and full reporting of outcomes
☒	☐	Estimates of effect sizes (e.g. Cohen's *d*, Pearson's *r*), indicating how they were calculated

Our web collection on statistics for biologists contains articles on many of the points above.

Software and code

Policy information about availability of computer code

Data collection	No codes were used for data collection.
Data analysis	Genome reconstruction (mapping, assembly) was performed using 454 software suite and SPAdes v3.13.1 51 (Bankevich et al. 2012). Sequence analysis was performed using Geneious Prime 2021.0.1; Biomatters, Auckland, New Zealand. Phylogenetic analysis was performed for complete-coding and partial seqeuences (N-X-P genes) using IQ-TREE software (version 2.2.2.6; Minh et al. 2020), based on a MUSCLE-Alignments (Edgar et al. 2004). Heatmap analysis was performed using pheatmap R-package (Kolde 2019). Root-to-tip distances were calculated from the ML tree of N-X/P sequences using TempEst (Rambaut et al. 2016). Isolation by distance analysis was perfomed using the “mantel” function of the “vegan” R-package. Geospatial data analysis and modelling was performed in R Studio (R v4.0.2) with the packages rnaturalearth (Massicotte et al. 2023) and ggplot2 (Wickham 2016). Non-parametric two-dimensional kernel density estimation was used to visualize spatial distribution patterns of mapped BoDV-1 cases, using “stat_density_2d” function in ggplot.

For manuscripts utilizing custom algorithms or software that are central to the research but not yet described in published literature, software must be made available to editors and reviewers. We strongly encourage code deposition in a community repository (e.g. GitHub). See the Nature Portfolio guidelines for submitting code & software for further information.

Data

Policy information about availability of data

All manuscripts must include a data availability statement. This statement should provide the following information, where applicable:

- Accession codes, unique identifiers, or web links for publicly available datasets
- A description of any restrictions on data availability
- For clinical datasets or third party data, please ensure that the statement adheres to our policy

All novel BoDV-1 sequences are available from the INSDC databases under accession numbers OR203629, OR203630, OR468838 to OR468971 (https://www.ncbi.nlm.nih.gov/nucleotide/). Accession numbers and metadata are listed in a separate file (Supplemental Data Set 1). BoDV-1 sequences of reanalysed previously published isolates (H640 and H3053) are available under accession numbers AY374523.2 and AY374537.2. Accession numbers of additional sequences derived from public databases are provided in the text and/or in the phylogenetic trees (Figure 2 and Supplemental Figure 5). Source data are provided with this paper.

Research involving human participants, their data, or biological material

Policy information about studies with human participants or human data. See also policy information about sex, gender (identity/presentation), and sexual orientation and race, ethnicity and racism.

Reporting on sex and gender	The sex of human patients was recorded as part of the data collection, but it was neither a criterion for selection of cases nor was data analysed in a sex-specific manner.
Reporting on race, ethnicity, or other socially relevant groupings	Neither race, ethnicity, or other socially relevant groupings were recorded for the human patients.
Population characteristics	Sex, age, year of disease, duration of disease (from hospitalization to death), location of last residence, viral loads in brain samples and sequence of the infecting BoDV-1 were recorded for human patients.
Recruitment	Diagnostic material from BoDV-1-infected humans were submitted by diagnostic laboratories, pathologists or clinicians. The samples originated from diagnostic testing as well as from restrospective screening of archived material. All cases of laboratory-confirmed human BoDV-1 infection (following the definition of Eisermann et al., 2019) known to the authors were included in the study.<br>Since direct virus detection (required for fulfilling the case definition criteria) may fail during intra vitam diagnosis, non-fatal BoDV-1 infection may be underrepresented among the studied cases. This has been discussed in the manuscript.
Ethics oversight	Ethical approval of the analysis of archived human samples was obtained from the local ethical commission of the Faculty for Medicine, University of Regensburg (ref. no. 18-1248-101), the Technical University Munich (577/19 S), the Ludwigs-Maximilians University Munich (23-0267) and the Medical Board of Hamburg (PV5616).

Note that full information on the approval of the study protocol must also be provided in the manuscript.

Field-specific reporting

Please select the one below that is the best fit for your research. If you are not sure, read the appropriate sections before making your selection.

☒ Life sciences ☐ Behavioural & social sciences ☐ Ecological, evolutionary & environmental sciences

For a reference copy of the document with all sections, see nature.com/documents/nr-reporting-summary-flat.pdf

Life sciences study design

All studies must disclose on these points even when the disclosure is negative.

Sample size	The sample size was not predetermined. Diagnostic laboratories were informed about the study through presentations at scientific meetings, publications in national specialist journals, via mailing lists of expert societies and by direct contact. All submitted cases were included in the study. Our dataset represents the largest and most comprehensive dataset on the natural occurrence of BoDV-1 infection in animals and humans that has been assembled so far.
Data exclusions	Duplicate sequences originating from the same individual as well as sequences previously identified as laboratory contaminants (see Dürrwald et al., 2006, 2007; Niller et al., 2020) were excluded from the analysis. GenBank-derived sequences not covering at least the N, X and P genes were likewise not included in the analysis.
Replication	All diagnostic methods were performed following established standard protocols, as described in the materials and methods section, and suitable positive and negative controls were employed to confirm the reliability of each assay. Detection of BoDV-1 RNA was performed by two RT-qPCR assays in parallel. Phylogenetic analyses were performed with the indicated numbers of bootstrap replicates and bootstrap values are provided in the figures. The outlier definition was performed based in parallel based on pairwise nucleotide identities and patrictic distances and both analyses provided consistent results.

Randomization	Samples/cases were not distributed into experimental groups.
Blinding	The technicians performing RNA extraction, RT-qPCR and sequencing were blinded to the identity of the samples.

Reporting for specific materials, systems and methods

We require information from authors about some types of materials, experimental systems and methods used in many studies. Here, indicate whether each material, system or method listed is relevant to your study. If you are not sure if a list item applies to your research, read the appropriate section before selecting a response.

Materials & experimental systems

n/a	Involved in the study
☒	☐ Antibodies
☒	☐ Eukaryotic cell lines
☒	☐ Palaeontology and archaeology
☐	☒ Animals and other organisms
☐	☒ Clinical data
☒	☐ Dual use research of concern
☒	☐ Plants

Methods

n/a	Involved in the study
☒	☐ ChIP-seq
☒	☐ Flow cytometry
☒	☐ MRI-based neuroimaging

Animals and other research organisms

Policy information about studies involving animals; ARRIVE guidelines recommended for reporting animal research, and Sex and Gender in Research

Laboratory animals	No laboratory animals were involved in this study.
Wild animals	Brain samples of BoDV-1-positive wild bicolored white-toothed shrews were obtained from an ongoing large-scale small mammal screening study (Haring et al., manuscript in preparation). Shrew KS20/0026 originated from a project that was commissioned by the Federal Environment Agency as part of the Environmental Research Plan (Research Code 3718 48 4250; animal ethics permit: 42502-2-1548 UniLeipzig). All other carcasses were found dead (e.g. preyed by cats or killed during pest control measures) and submitted for further analysis. No samples were collected from life wild animals.
Reporting on sex	Sex of animals was not recorded as part of this study.
Field-collected samples	Animal samples included brain samples collected post mortem during diagnostic necropsy. Housing conditions of the diseased animals were not recorded.
Ethics oversight	Shrew KS20/0026 originated from a project that was commissioned by the Federal Environment Agency as part of the Environmental Research Plan (Research Code 3718 48 4250; animal ethics permit: 42502-2-1548 UniLeipzig).<br>No ethics oversight was required for analysis of diagnostic samples collected post mortem during necropsy.

Note that full information on the approval of the study protocol must also be provided in the manuscript.

Clinical data

Policy information about clinical studies

All manuscripts should comply with the ICMJE guidelines for publication of clinical research and a completed CONSORT checklist must be included with all submissions.

Clinical trial registration	The study does not report a clinical trial.
Study protocol	*Note where the full trial protocol can be accessed OR if not available, explain why.*
Data collection	*Describe the settings and locales of data collection, noting the time periods of recruitment and data collection.*
Outcomes	*Describe how you pre-defined primary and secondary outcome measures and how you assessed these measures.*

Plants

Seed stocks

Plants were not part of the study.

Novel plant genotypes

Describe the methods by which all novel plant genotypes were produced. This includes those generated by transgenic approaches, gene editing, chemical/radiation-based mutagenesis and hybridization. For transgenic lines, describe the transformation method, the number of independent lines analyzed and the generation upon which experiments were performed. For gene-edited lines, describe the editor used, the endogenous sequence targeted for editing, the targeting guide RNA sequence (if applicable) and how the editor was applied.

Authentication

Describe any authentication procedures for each seed stock used or novel genotype generated. Describe any experiments used to assess the effect of a mutation and, where applicable, how potential secondary effects (e.g. second site T-DNA insertions, mosiacism, off-target gene editing) were examined.
